# Mitochondrial Fission Is Required for Blue Light-Induced Apoptosis and Mitophagy in Retinal Neuronal R28 Cells

**DOI:** 10.3389/fnmol.2018.00432

**Published:** 2018-11-27

**Authors:** Jia-Yu Li, Kun Zhang, Dan Xu, Wen-Tian Zhou, Wen-Qing Fang, Yu-Ying Wan, Dan-Dan Yan, Miao-Yu Guo, Jin-Xin Tao, Wen-Chuan Zhou, Fan Yang, Li-Ping Jiang, Xiao-Jian Han

**Affiliations:** ^1^Research Institute of Ophthalmology and Visual Sciences, Affiliated Eye Hospital of Nanchang University, Nanchang, China; ^2^National Engineering Technology Research Center for LED on Silicon Substrate, Nanchang University, Nanchang, China; ^3^Department of Intra-Hospital Infection Management, The Second Affiliated Hospital of Nanchang University, Nanchang, China; ^4^Department of Pharmacology, School of Pharmaceutical Science, Nanchang University, Nanchang, China

**Keywords:** blue light, retinal neuronal cells, apoptosis, mitochondrial fission, mitophagy

## Abstract

Light emitting diodes (LEDs) are widely used to provide illumination due to their low energy requirements and high brightness. However, the LED spectrum contains an intense blue light component which is phototoxic to the retina. Recently, it has been reported that blue light may directly impinge on mitochondrial function in retinal ganglion cells (RGCs). Mitochondria are high dynamic organelles that undergo frequent fission and fusion events. The aim of our study was to elucidate the role of mitochondrial dynamics in blue light-induced damage in retinal neuronal R28 cells. We found that exposure to blue light (450 nm, 1000 lx) for up to 12 h significantly up-regulated the expression of mitochondrial fission protein Drp1, while down-regulating the expression of mitochondrial fusion protein Mfn2 in cells. Mitochondrial fission was simultaneously stimulated by blue light irradiation. In addition, exposure to blue light increased the production of reactive oxygen species (ROS), disrupted mitochondrial membrane potential (MMP), and induced apoptosis in R28 cells. Notably, Drp1 inhibitor Mdivi-1 and Drp1 RNAi not only attenuated blue light-induced mitochondrial fission, but also alleviated blue light-induced ROS production, MMP disruption and apoptosis in cells. Compared with Mdivi-1 and Drp1 RNAi, the antioxidant N-acetyl-L-cysteine (NAC) only slightly inhibited mitochondrial fission, while significantly alleviating apoptosis after blue light exposure. Moreover, we examined markers for mitophagy, which is responsible for the clearance of dysfunctional mitochondria. It was found that blue light stimulated the conversion of LC3B-I to LC3B-II as well as the expression of PINK1 in R28 cells. Mdivi-1 or Drp1 RNAi efficiently inhibited the blue light-induced expression of PINK1 and co-localization of LC3 with mitochondria. Thus, our data suggest that mitochondrial fission is required for blue light-induced mitochondrial dysfunction and apoptosis in RGCs.

## Introduction

The revolution in lighting technologies has dramatically changed human life style while leading to the excessive exposure of human eyes to artificial light. With their minimal energy requirements and high brightness, light-emitting diodes (LEDs) are not only widely used for general illumination, but have also become the dominant technology for backlit tablet displays. However, LEDs exhibit a peak emission in the blue light range (400–490 nm) (O’Hagan et al., 2016). Emerging evidence indicates that blue light can regulate circadian rhythms, sleeping patterns and other biological functions (Tosini et al., 2016). In mammals, there are three types of photoreceptors in the retina, including cones, rods, and the intrinsically photosensitive RGCs (ipRGCs). Melanopsin exclusively expressed in ipRGCs is the most likely candidate as the circadian retinal photo pigment with an absorption peak around 470–480 nm, which is involved in the modulation of circadian rhythms, sleep, mood, and learning (Hattar et al., 2002; Bailes and Lucas, 2013). Alternatively, several studies indicate that blue light is also phototoxic to the retina. Excessive exposure to blue light may induce severe cellular damage in the retina, including photoreceptors, retinal pigment epithelial cells, and RGCs (Argun et al., 2014; Del Olmo-Aguado et al., 2016; Kim et al., 2016).

The wavelength of light reaching the retina is mainly in the range of 400–780 nm after absorption by the cornea, lens, and vitreous (Osborne et al., 2014). Interestingly, the axons of RGCs in the ocular globe are mostly unmyelinated and rich in mitochondria. As a result, blue light may impinge on the intra-axonal mitochondria of ganglion cells before penetrating to the deeper retinal cells (Osborne, 2008; Del Olmo-Aguado et al., 2012). Moreover, mitochondrial chromophores including cytochrome oxidase, cytochrome P450, and flavin constituents mostly absorb light with wavelengths in the blue light range (Ortiz de Montellano, 1995; García and Silva, 1997; Osborne et al., 2014). The interaction between blue light and mitochondrial chromophores may induce oxidative stress via photochemical or photodynamic effects (Tosini et al., 2016). Recently, it has been reported that blue light induces damage to retinal ganglion cells by its direct action on mitochondria (Osborne et al., 2006, 2008; Knels et al., 2011; Del Olmo-Aguado et al., 2012). The light insult decreases the metabolic state of isolated mitochondria via direct action on dehydrogenase and mitochondrial complexes I–V (Osborne et al., 2006; Del Olmo-Aguado et al., 2012). Light insult also changes mitochondrial morphology in RGC-5 and R28 cells from elongated into dot-like shapes (Knels et al., 2011; Del Olmo-Aguado et al., 2012). Furthermore, mitochondria are high dynamic organelles that undergo frequent fission and fusion events (Suárez-Rivero et al., 2016). The process of mitochondrial fission is mediated by dynamin-related protein 1 (Drp1), while mitofusin 1/2 and OPA1 act as the mediators of mitochondrial outer or inner membrane fusion in mammalian cells, respectively (Lee and Yoon, 2016). Under stresses or apoptotic stimulation, the balance of mitochondrial dynamics may be shifted to excessive fission, thereby leading to both mitochondrial and cellular damage (Suen et al., 2008). In cells, an important quality control system exists to remove the seriously damaged mitochondria via mitophagy (Rüb et al., 2017). The PINK1/parkin system acts as a sensor for mitochondrial quality and is activated by the loss of mitochondrial membrane potential (MMP) (Matsuda et al., 2010; Narendra et al., 2010). In addition, the specific removal of individual damaged mitochondria seems to be supported by a fission process that separates the healthy from the defective organelles (Youle and van der Bliek, 2012). However, whether blue light induces apoptosis in RGCs by promoting mitochondrial fission, the disruption of MMP and activation of mitophagy remains to be further elucidated.

In the present study, we found that blue light at 450 nm stimulated the expression of mitochondrial fission protein Drp1 and down-regulated fusion protein Mfn2 in R28 cells. Simultaneously, pronounced mitochondrial fission was induced by blue light insult. Inhibition of mitochondrial fission by pretreatment with Mdivi-1 or Drp1 RNAi efficiently attenuated blue light-induced ROS production, disruption of MMP, and apoptosis in R28 cells. Furthermore, blue light also stimulated the conversion of autophagy marker LC3B-I to LC3B-II and up-regulated the expression of PINK1. Inhibition of mitochondrial fission by Mdivi-1 or Drp1 RNAi efficiently inhibited the blue light-induced expression of PINK1 and co-localization of LC3 with mitochondria. This suggests that Drp1-mediated mitochondrial fission after blue light insult activates the removal of dysfunctional mitochondria via mitophagy. Therefore, our studies indicate the direct action of blue light on mitochondrial dynamics; they also assert that excessive mitochondrial fission is required for apoptosis in RGCs after blue light irradiation.

## Experimental Procedures

### Cell Culture

R28 cells were kindly provided by Guo-Tong Xu (Tongji Eye Institute, Tongji University School of Medicine; China Stem Cell Bank East China Bank, Shanghai, China). As described previously (Gu et al., 2014), the cells were grown in low glucose DMEM (BI, Beit HaEmek, Israel) supplemented with 10% fetal bovine serum (BI, Beit HaEmek, Israel) and 1% penicillin-streptomycin (Solarbio, Beijing, China) and maintained in a humidified incubator at 37°C with an atmosphere containing 5% CO_2_.

### Light Exposure

To detect their response to blue light, R28 cells were treated with three different exposure conditions, including darkness, blue light at 450 nm, and red light at 650 nm. Spectral irradiances at the area of exposure were measured using a digital spectroradiometer (TASI-8720, TASI Electronics Co., Ltd., Suzhou, China). For light exposure, cells plated in 35 mm dishes were irradiated by blue light (450 nm, 1000 lx) and red light (630 nm, 1000 lx) for different time periods (varying from 0 to 24 h). To prevent Drp1-mediated mitochondrial fission, R28 cells were pretreated with 5 μM of Mdivi-1 or transfected with Drp1 siRNA 2 or 48 h prior to irradiation. Non-irradiated cells grown in a conventional cell incubator in the dark served as controls. The temperature of the incubator was maintained at 37°C during irradiance with blue or red light up to 24 h.

### Immunofluorescence Staining

R28 cells were transfected with pDsRed2-Mito to label mitochondria. Twenty-four hours later, cells were exposed to light irradiation for 18 h. After irradiation, cells were fixed in 4% ice-cold paraformaldehyde (PFA) for 10 min and washed three times with PBS. Fixed cells were permeabilized with 0.5% Triton-X 100 and 0.5% bovine serum albumin (BSA) in PBS at room temperature for 30 min. For immunofluorescence staining, cells were incubated with polyclonal rabbit anti-LC3 (ab51520, 1:200; Abcam, Cambridge, MA, United States) at 4°C overnight and washed three times with 0.5% Triton-X 100 in PBS. Then cells were incubated with Alexa Fluor 488-conjugated AffiniPure Goat anti-Rabbit IgG (SA00006-2, 1:200; Proteintech, Rosemont, IL, United States) at room temperature in the dark for 2 h. Both primary and secondary antibodies were diluted in PBS with 0.5% Triton-X 100 and 0.5% BSA. Fluorescence signals were observed using a confocal microscope (LSM 800; Carl Zeiss Microscopy, Jena, Germany).

### Detection of Intracellular ROS

The intracellular and mitochondrial ROS levels in R28 cells were measured by detecting the fluorescence signal of 2′,7′-dichlorodihydrofluorescein diacetate (DCFH-DA, Sigma-Aldrich, St. Louis, MO, United States) and MitoSOX (Invitrogen, Thermo Fisher Scientific, Carlsbad, CA, United States), respectively. First, the intracellular ROS level at 6–24 h after blue light irradiation was examined. Cells cultured in the dark or exposed to red light were used as controls or negative controls, respectively. According to the manufacturer’s instructions, cells were incubated with 10 μM of DCFH-DA for 30 min at 37°C. To detect mitochondrial ROS levels, cells were incubated with 5 μM MitoSOX for 10 min at 37°C in the dark. Then the cells stained with DCFH-DA or MitoSOX were washed twice with PBS or HBSS/Ca^2+^/Mg^2+^ wash buffer, respectively. The fluorescence signals of DCFH-DA and MitoSOX were observed under a fluorescence microscope (IX71, Olympus, Tokyo, Japan). To investigate the effect of Drp1-mediated mitochondrial fission on irradiation-induced intracellular ROS production, cells were pretreated with 5 μM of Mdivi-1 2 h prior to blue light exposure or transfected with Drp1 siRNA 48 h prior to blue light exposure. In addition, the cells were harvested after MitoSOX staining and MitoSOX-positive cells were counted by flow cytometry (FACS Canto^®^II, BD Biosciences, San Jose, CA, United States) with the excitation source at 510 nm and emission wavelength of 580 nm. The flow cytometry data were analyzed using FlowJO^TM^ software (BD Biosciences).

### Measurement of Mitochondrial Membrane Potential (Δψm)

Mitochondrial membrane potential (Δψm) was measured by detecting the fluorescence signals of tetramethylrhodamine ethyl ester (TMRE, Invitrogen). Cells cultured in the dark or exposed to red right were used as controls or negative controls, respectively. To investigate the role of mitochondrial fission in irradiation-induced alteration in Δψm, R28 cells were pretreated with 5 μM of Mdivi-1 2 h prior to blue light irradiation. According to the manufacturer’s instructions, cells were incubated with 50 nM of TMRE for 20 min at 37°C, then washed twice with PBS. The TMRE fluorescence signals were observed under a fluorescence microscope (IX71, Olympus). In addition, the cells were harvested after TMRE staining and TMRE-positive cells were counted by flow cytometry (FACS Canto^®^ II, BD) with the excitation source at 543 nm and emission wavelength of 560 nm. The flow cytometry data were analyzed using FlowJO^TM^ software (BD Biosciences).

### Transfection Procedures

To label mitochondria, R28 cells were transfected with pDsRed2-Mito. As described previously (Guo et al., 2018), the transfection procedure was performed according to the manufacturer’s instructions for Lipofectamine 2000^®^(Invitrogen). After 6 h of incubation, the medium containing Lipofectamine was replaced with normal culture medium. The transfection efficiency of cells was further confirmed under a fluorescence microscope (IX71, Olympus) 1 day later.

### Western Blot Analysis

After twice washes with PBS, R28 cells were harvested in lysis buffer A [20 mM Tris (pH 7.4), 150 mM NaCl, 10 mM sodium orthovanadate, 20 mM sodium fluoride, 0.25 M sucrose, 1 mM dithiothreitol, 500 nM okadaic acid, and 0.5% Tween 20] and incubated in lysis buffer for 30 min on ice. Then, whole cell lysates were subjected to sonication in 4 × sample buffer, and boiled for 5 min. Proteins in lysates were separated by SDS–PAGE gel electrophoresis, and further transferred onto PVDF membrane. After blocking with 5% skim milk, proteins on membranes were immunoblotted overnight at 4°C with the following primary antibodies: DRP1 Rabbit mAb (#8570, 1:1000; Cell Signaling Technology, Danvers, MA, United States), Phospho-DRP1 Antibody (#3455, 1:1000; Cell Signaling Technology), Mitofusin-2 Rabbit mAb (#9482, 1:1000; Cell Signaling Technology), OPA-1 (#80471, 1:1000; Cell Signaling Technology), cleaved caspase 3 (#9664, 1:1000; Cell Signaling Technology), Anti-LC3B antibody (ab51520, 1:3,000; Abcam, United States), PINK1 (#6946, 1:1000; Cell Signaling Technology), β-actin (#4967S, 1:5000; Cell Signaling Technology). After three washes in TBST, the membranes were incubated with HRP-conjugated secondary antibody (CW0103T, 1:5000; CW Biotech, Beijing, China) for 1 h at room temperature. Western blot bands were detected using an enhanced chemiluminescence solution (CW Biotech). Densitometric analysis was performed using ImageJ software (NIH, Bethesda, MD, United States).

### RNA Interference

For selective knockdown of Drp1, three pairs of Drp1-siRNA and a scramble RNA were designed according to a rat Drp1 gene transcript (NCBI GenBank accession number NC_005110.4; NIH) and chemically synthesized by Biomics Biotechnologies Co., Ltd. (Beijing, China). The nucleotide sequences of RNA were as follows: Dnm1l-si-1 5′-GGAACAAAGUAUCUUGCUAdTdT-3′ (sense) and 5′-UAGCAAGAUACUUUGUUCCdTdT-3′ (antisense), Dnm1l-si-2 5′-GUAUCGCGAGACAAGUUAAdTdT-3′ (sense) and 5′-UUAACUUGUCUCGCGAUACdTdT-3′ (antisense), Dnm1l-si-3 5′-CGCUGAUCCCGGUCAUCAAdTdT-3′ (sense) and 5′-UUGAUGACCGGGAUCAGCGdTdT-3′ (antisense), respectively. A pair of scramble RNA with nucleotide sequences of 5′-UUCUCCGAACGUGUCACGUdTdT-3′ (sense) and 5′-ACGUGACACGUUCGGAGAAdTdT-3′ (antisense) was used as negative controls. siRNAs were transfected into R28 cells using Lipofectamine 2000^®^(Invitrogen) according to the manufacturer’s instructions. Drp1 expression was further examined by western blotting to evaluate the silencing efficiency 48 h after transfection.

### Terminal Deoxynucleotidyl Transferase dUTP Nick End Labeling (TUNEL) Assay

Apoptosis in R28 cells after blue light irradiation was examined by TUNEL assay. Cells cultured in the dark or exposed to red right were used as controls or negative controls, respectively. To examine the role of mitochondrial fission and ROS in apoptosis, cells were incubated with Mdivi-1 or the antioxidant N-acetyl-L-cysteine [NAC (Sigma-Aldrich); 200 μM, 1 h prior to irradiation], or transfected with Drp1 siRNA before blue light irradiation. According to the manufacturer’s instructions (Roche, Indianapolis, IN, United States), R28 cells were gently washed with PBS after blue light irradiation, and incubated with permeabilization solution containing 0.1% Triton X-100 at room temperature for 5 min. Then cells were further incubated with terminal deoxynucleotidyl transferase at 37°C for 1 h and stained with dUTP-fluorescein isothiocyanate. Moreover, cell nuclei were counterstained with DAPI. The fluorescent signals were visualized under a fluorescence microscope (IX71, Olympus), and cells with TUNEL-positive nuclei were counted as apoptotic cells.

### Mitochondrial Imaging and Analysis

As described previously (Han et al., 2008; Guo et al., 2018), pDsRed2-Mito was transfected into R28 cells to label mitochondria with lipofectamine 2000^®^ (Invitrogen). To examine the role of Drp1 and ROS in blue light-induced mitochondrial fission, cells were incubated with Mdivi-1 or the antioxidant NAC, or transfected with Drp1 siRNA before blue light irradiation. Cells cultured in the dark or exposed to red right were used as controls or negative controls, respectively. Mitochondrial imaging was acquired using a Zeiss confocal microscope (LSM 800). Mitochondrial length was measured by tracing the fluorescence signals of DsRed2 using ZEN 2.3SP1 developed by Carl Zeiss Microscopy. The average value of mitochondrial length in each group was measured and analyzed. For each group, approximately 270 mitochondria from 5 to 8 different random fields were measured.

### Statistical Analysis

All results are presented as mean ± standard deviation. Statistical analysis of data was performed using the SPSS 23.0 statistical package (IBM Corp., Armonk, NY, United States). Multiple comparisons were analyzed using one-way ANOVA followed by the Dunnett-*t* test or Tukey test for data with variance homogeneity and a normal distribution according to the applications. Differences with *p* < 0.05 were considered statistically significant.

## Results

### Blue Light Irradiation Induces Alterations in Mitochondrial Fission and Fusion Proteins

**FIGURE 1:**
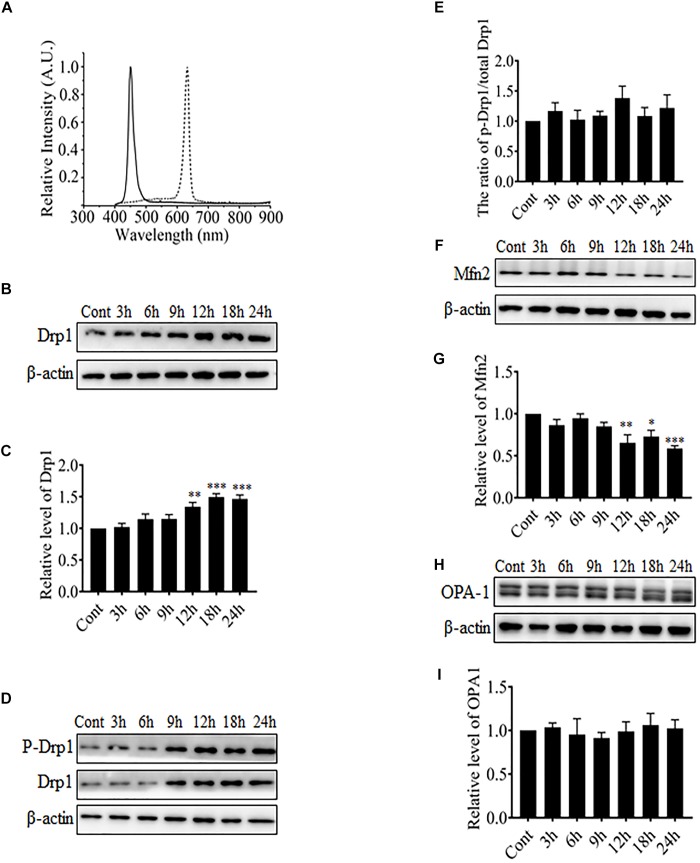
The effect of blue light on mitochondrial dynamics-related proteins. **(A)** The spectra of blue light (solid line) and red light (dotted line) used in this study. **(B,D,F,H)** After blue light irradiation for the indicated durations, R28 cells were harvested for western blot analysis to detect the expression level of Drp1 **(B)**, Mfn2 **(F)**, and OPA-1 **(H)**. Cells cultured in the dark were used as controls. Moreover, the phosphorylation of Drp1 at Ser616 was also examined **(D)**. β-actin was used as an endogenous control. **(C,E,G,I)** The graphs indicate the normalization of the levels of Drp1 **(C)**, phosph-Drp1 **(E)**, Mfn2 **(G)**, and OPA-1 **(I)** in cells after blue light exposure at indicated time points. Relative expression level of each protein is indicated as a normalization of the ratio of mitochondrial dynamics-related protein/β-actin in each sample to the control. Data presented as the mean ± SD of at least three independent experiments (*n* = 5 in **C** and **I**, *n* = 4 in **E** and **G**). ^∗^*p* < 0.05; ^∗∗^*p* < 0.01; ^∗∗∗^*p* < 0.001 vs. Control. In contrast, red light did not influence the expression of mitochondrial dynamics-related protein (please refer to Supplementary Figures 1A–F).

It was reported in previous studies that mitochondria in RGCs were changed from elongated thread-like shapes into dot-like shapes after exposure to blue light, which is very similar to changes observed during mitochondrial fission (Knels et al., 2011; Del Olmo-Aguado et al., 2012). Mitochondrial morphology is regulated by certain highly conserved large GTPases including Drp1, Mfn1 and 2, and OPA-1. Drp1 is required for mitochondrial fission, whereas Mfn1 and 2 and OPA-1 mediate the fusion of outer mitochondrial membranes and inner mitochondrial membranes, respectively. Here, the R28 cells were exposed to blue light at 450 nm or red light at 630 nm. The spectral irradiation data for blue or red light are shown in Figure 1A. After light irradiation, the expression levels of mitochondrial fission or fusion proteins in R28 cells were examined using western blot analysis. It was found that red light irradiation did not induce any significant alterations in the expression or phosphorylation of mitochondrial fission or fusion proteins including Drp1 and Mfn2 (Supplementary Figures 1A–F). In contrast, Drp1 expression was significantly up-regulated after exposure to blue light for up to 12–24 h (Figures 1B,C). As the phosphorylation at Ser616 is important for the GTPase activity of Drp1 and mitochondrial fission (Taguchi et al., 2007), we further examined the phosphorylation of Drp1 at Ser616 after blue light irradiation. As shown in Figures 1D,E, the phosphorylation level of Drp1 at Ser616 was not significantly altered after blue light irradiation. In contrast, the expression of mitochondrial outer membrane fusion protein Mfn2 was significantly down-regulated after blue light exposure for up to 12–24 h (Figures 1F,G), whereas there was no obvious alteration in OPA-1 expression (Figures 1H,I). Moreover, these results suggest that blue light might stimulate mitochondrial fission through alterations in the expression of mitochondrial dynamics-related proteins in R28 cells.

### Blue Light Shifts Mitochondrial Dynamics to Fission in R28 Cells

**FIGURE 2:**
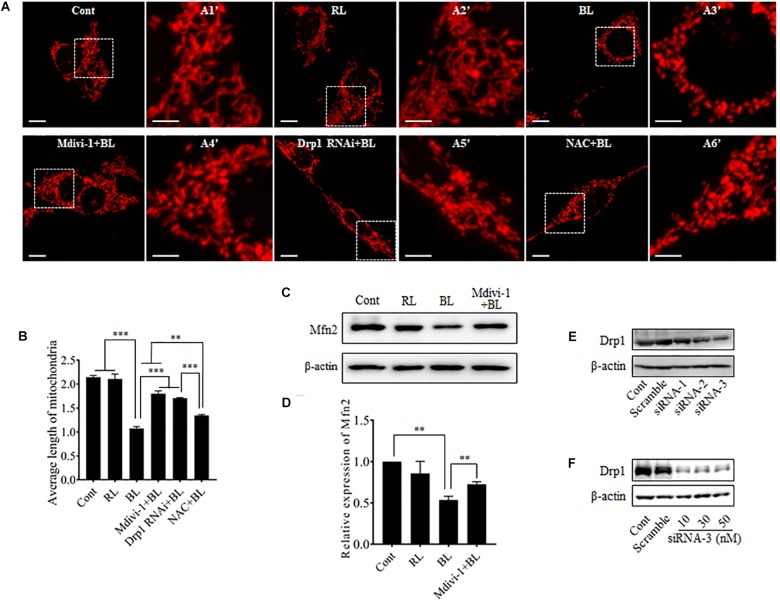
Effect of Mdivi-1, Drp1 RNAi, and NAC on blue light-induced mitochondrial fission and Mfn2 expression. **(A)** Mitochondrial morphology in R28 cells irradiated with or without blue light. Cells were transfected with pDsRed2-Mito to label mitochondria. The fluorescence of mitochondria-targeted DsRed was highly tolerant to prolonged blue light irradiation (please refer to Supplementary Figure 2). The fluorescence signal of pDsRed2-Mito indicated mitochondrial morphology in cells, scale bar = 10 μm. A1′, A2′, A3′, and A4′ show mitochondria with higher magnification in the inserted boxes, scale bar = 5 μm. **(B)** Effect of Mdivi-1, Drp1 RNAi, and NAC on blue light-induced mitochondrial fission. The average length of mitochondria in each group was measured. The data is presented as the mean ± SD of three independent experiments. 270 mitochondria/group were analyzed. The unit of average length of mitochondria is μm; ^∗∗^*p* < 0.01, ^∗∗∗^*p* < 0.001. **(C)** Expression level of Mfn2 was immunoblotted in R28 cells after the indicated treatments. β-actin was used as an endogenous control. **(D)** Relative expression level of Mfn2 was indicated as a normalization of the ratio of Mfn2/β-actin in each sample to the control. The data are presented as the mean ± SD of four independent experiments; ^∗∗^*p* < 0.01; Cont, control; RL, red light; BL, blue light. **(E)** Silencing efficiency of different siRNAs on Drp1 in R28 cells. Expression level of Drp1 was immunoblotted in R28 cells transfected with siRNA-1∼3 or scramble RNA. Cells without transfection were used as controls. **(F)** Silencing efficiency of siRNA-3 at 10∼50 nM on Drp1 expression in R28 cells.

To investigate the role of blue light-induced Drp1 up-regulation in mitochondrial dynamics, we observed mitochondrial morphology in cells under a confocal microscope. pDsRed2-Mito was transfected into R28 cells for mitochondrial imaging. First, we examined the photobleaching effect of prolonged blue light irradiation on mitochondria-targeted DsRed. As shown in Supplementary Figure 2, the fluorescence of mitochondria-targeted DsRed was highly tolerant to blue light irradiation for up to 24 h. Consistent with previous studies (Knels et al., 2011; Del Olmo-Aguado et al., 2012), mitochondria in R28 cells without irradiation appeared as tubular, thread-like networks and red light irradiation did not induce obvious alterations in mitochondrial morphology (Figure 2A). However, blue light irradiation for 12 h significantly reduced the average length of mitochondria and induced the fragmented, punctate dot-like mitochondria (Figures 2A,B). Interestingly, mitochondria in R28 cells pretreated with 5 μM Drp1 inhibitor Mdivi-1 retained the average length and appeared as elongated tubular, thread-like networks after irradiation (Figure 2B). Mdivi-1 also alleviated the blue light-induced down-regulation of Mfn2 (Figures 2C,D). To rule out potential off-target effects of Mdivi-1, we performed siRNA experiments to silence Drp1 in cells. As shown in Figures 2E,F, siRNA-3 at 30 nM stably silenced the expression of Drp1 in R28 cells. As expected, knockdown of Drp1 significantly attenuated blue light-induced mitochondrial fission (Figures 2B,C). Moreover, the effect of antioxidant NAC on blue light-induced mitochondrial fission was also examined. It was found that the efficiency of NAC was much weaker than Mdivi-1 or Drp1 RNAi, although NAC also alleviated blue light-induced mitochondrial fission (Figures 2B,C). These results indicate that blue light irradiation stimulates mitochondrial fission which can be efficiently prevented by Mdivi-1 and Drp1 RNAi in R28 cells.

### Inhibition of Mitochondrial Fission Protects Cells Against Blue Light-Induced Apoptosis

**FIGURE 3:**
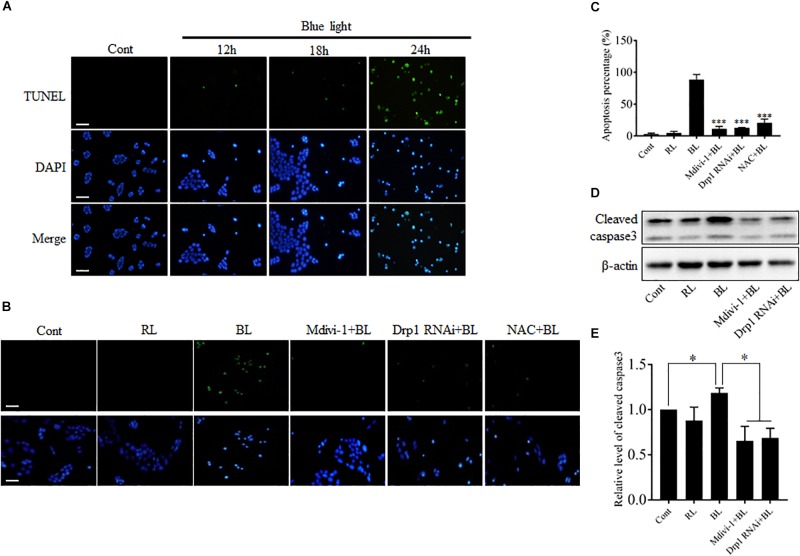
The apoptosis and cleavage of caspase 3 induced by blue light were alleviated by Mdivi-1, Drp1 RNAi, and NAC. **(A)** The apoptosis of R28 cells after blue light irradiation. Cells were stained with TUNEL to detect cellular apoptosis and counterstained with DAPI. Scale bar = 25 μm. Red light irradiation did not induce obvious apoptosis in R28 cells (please refer to Supplementary Figure 4). **(B,C)** Effect of Mdivi-1, Drp1 RNAi, and NAC on blue light-induced apoptosis in R28 cells. Scale bar = 25 μm. The graph indicates the apoptosis percentage in each group. 300 cells/group were analyzed, *n* = 3; ^∗∗∗^*p* < 0.001 vs. BL. **(D)** The level of cleaved caspase 3 was detected by western blot analysis after the indicated treatments. β-actin was used as an endogenous control. **(E)** Relative level of cleaved caspase 3 was indicated as a normalization of the ratio of cleaved caspase 3/β-actin in each sample to the control. The data are presented as the mean ± SD of four independent experiments; ^∗^*p* < 0.05; Cont, control; RL, red light; BL, blue light.

Blue light-induced injuries have been reported in retinal photoreceptor cells, pigment epithelium cells, and ganglion cells (Argun et al., 2014; Del Olmo-Aguado et al., 2016; Kim et al., 2016). Here, we further examined the effect of blue light at 450 nm on the survival of R28 cells using double staining with TUNEL and DAPI. As shown in Figure 3A, few apoptotic cells were present in control group and groups with blue light irradiation for 12 and 18 h, whereas blue light exposure for 24 h induced obvious apoptosis in R28 cells. To investigate the role of Drp1-mediated mitochondrial fission in blue light-induced apoptosis, R28 cells were pretreated with 5 μM Mdivi-1 or transfected with Drp1 siRNA. It was found that red light irradiation had no obvious effect on the survival of R28 cells (Supplementary Figure 4), which is consistent with a previous study (Knels et al., 2011). Importantly, pretreatment with Mdivi-1, NAC, and Drp1 RNAi efficiently attenuated blue light-induced apoptosis in R28 cells (Figures 3B,C). Activation of caspase 3 was reported in the blue light-induced mitochondria-associated intrinsic apoptosis pathway (Knels et al., 2011; Huang et al., 2014). In agreement with this, the level of cleaved caspase 3 in cells after blue light irradiation was markedly higher than that in the control group and red light (RL) group. Pretreatment with Mdivi-1 or Drp1 RNAi efficiently attenuated blue light-induced cleavage of caspase 3 in R28 cells (Figures 3D,E). These results suggest the important role of Drp1-mediated mitochondrial fission in the blue light-induced intrinsic apoptosis pathway in R28 cells.

**FIGURE 4:**
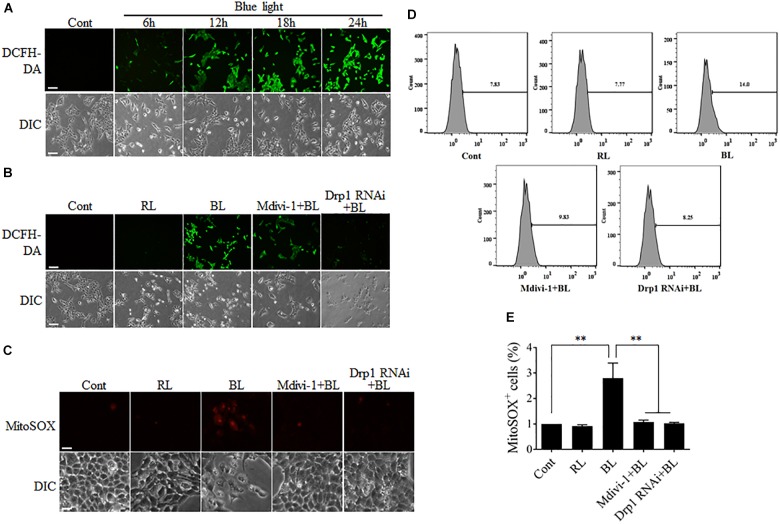
ROS production after blue light irradiation was attenuated by Mdivi-1 or Drp1 RNAi. **(A)** Blue light increased the intracellular ROS level in a time-dependent manner. R28 cells were cultured in the dark (control) or exposed to blue light for the indicated duration. The fluorescence signals of DCFH-DA were detected as intracellular ROS under a fluorescence microscope. Scale bar = 50 μm. **(B)** Effect of Mdivi-1 and Drp1 RNAi on blue light-induced intracellular ROS in R28 cells. The fluorescence signals of DCFH-DA were detected as the intracellular ROS in cells after the indicated treatments under a fluorescence microscope. Cont, control; RL, red light (also refer to Supplementary Figure 3A); BL, blue light. Scale bar = 50 μm. **(C)** Effect of Mdivi-1 and Drp1 RNAi on blue light-induced mitochondrial ROS in R28 cells. The fluorescence signals of MitoSOX in cells after the indicated treatments were detected under a fluorescence microscope. Scale bar = 20 μm. **(D)** After the indicated treatments, the mitochondrial ROS levels were measured by flow cytometry using MitoSOX staining. **(E)** The percentage of MitoSOX-positive cells in each group was measured. The data are presented as the mean ± SD of four independent experiments; ^∗∗^*p* < 0.01.

### Blue Light Increases Intracellular ROS Level Through the Mitochondrial Pathway

Although the sources of intracellular ROS include the mitochondrial electron transport chain (ETC), cyclooxygenases, xanthine oxidases, lipoxygenases, and NADPH oxidases, electron leak from the mitochondrial ETC is the major source of ROS (Abramov et al., 2007). It has been reported that mitochondrial dynamics is also relevant to the maintenance of intracellular ROS level (Willems et al., 2015). Disruption of mitochondrial dynamics may lead to impairment of mitochondrial functions, thereby influencing intracellular ROS production. To investigate the underlying mechanism of mitochondrial fission in blue light-induced apoptosis, the intracellular ROS levels in R28 cells were measured by detection of the fluorescence signals of DCFH-DA using a microscope. As shown in Figure 4A, the number of cells with strong green fluorescence of DCFH-DA was increased dramatically in a time-dependent manner after blue light irradiation. In contrast, red light did not induce the intense fluorescence signals of DCFH-DA in cells (Supplementary Figure 3A). Pretreatment with Mdivi-1 or Drp1 RNAi efficiently weakened the fluorescence of DCFH-DA in cells after blue light exposure (Figure 4B). Moreover, mitochondrial ROS levels in R28 cells were measured by detection of the fluorescence signals of MitoSOX using a microscope or flow cytometry. As shown in Figure 4C, blue light irradiation induced the strong fluorescence of MitoSOX in R28 cells. Pretreatment with Mdivi-1 or Drp1 RNAi weakened the fluorescence of MitoSOX in cells after blue light exposure. As expected, the results of flow cytometry also showed that blue light exposure for 12 h increased the percentage of MitoSOX-positive cells when compared to control and red light groups. Mdivi-1 and Drp1 RNAi significantly reduced the percentage of MitoSOX-positive cells induced by blue light irradiation (Figures 4D,E). These results suggest that mitochondrial fission plays an important role in the increase of intracellular and mitochondrial ROS after blue light exposure.

### Mitochondrial Fission After Blue Light Irradiation Disrupts Mitochondrial Membrane Potential

**FIGURE 5:**
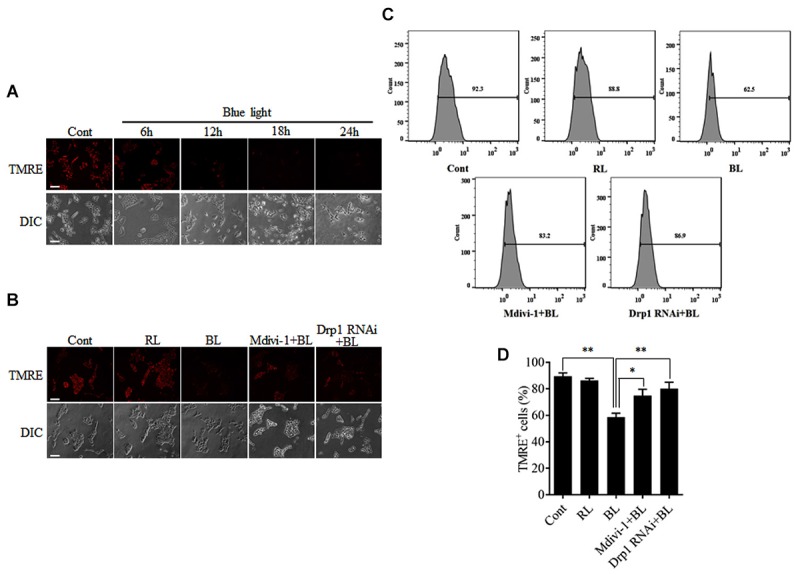
Effect of Mdivi-1 and Drp1 RNAi on blue light-induced disruption of mitochondrial membrane potential in R28 cells. **(A)** Blue light decreased the mitochondrial membrane potential in a time-dependent manner. R28 cells were cultured in the dark (control) or exposed to blue light for the indicated duration. The fluorescence signals of TMRE were detected as mitochondrial membrane potential under a fluorescence microscope. Scale bar = 50 μm. **(B)** Effect of Mdivi-1 and Drp1 RNAi on blue light-induced disruption in mitochondrial membrane potential in R28 cells. The fluorescence signals of TMRE in cells after the indicated treatments were detected under a fluorescence microscope. Cont, control; RL, red light (also refer to Supplementary Figure 3B); BL, blue light. Scale bar = 50 μm. **(C)** After the indicated treatments, the mitochondrial membrane potential was measured by flow cytometry using TMRE staining. **(D)** The percentage of TMRE-positive cells in each group was measured. The data are presented as the mean ± SD of four independent experiments; ^∗^*p* < 0.05; ^∗∗^*p* < 0.01.

To examine the functional consequence of blue light-induced mitochondrial fission, MMP was measured by detection of the fluorescence signals of TMRE using a microscope or flow cytometry. As shown in Figure 5A, blue light exposure markedly decreased the fluorescence of TMRE in R28 cells in a time-dependent manner. However, red light had no obvious effect on the TMRE fluorescence signals in cells (Supplementary Figure 3B). Pretreatment with 5 μM Mdivi-1 or Drp1 RNAi efficiently prevented the blue light-induced decrease in the fluorescence of TMRE in cells (Figure 5B). The results of flow cytometry also showed that blue light exposure for 12 h decreased the percentage of TMRE-positive cells when compared to control and red light groups. Mdivi-1 and Drp1 RNAi significantly inhibited the decrease in the percentage of TMRE-positive cells induced by blue light irradiation (Figures 5C,D). These results suggest that Drp1-mediated mitochondrial fission is involved in the disruption of MMP in R28 cells after blue light exposure.

### Mitochondrial Fission After Blue Light Exposure Triggers Mitophagy

**FIGURE 6:**
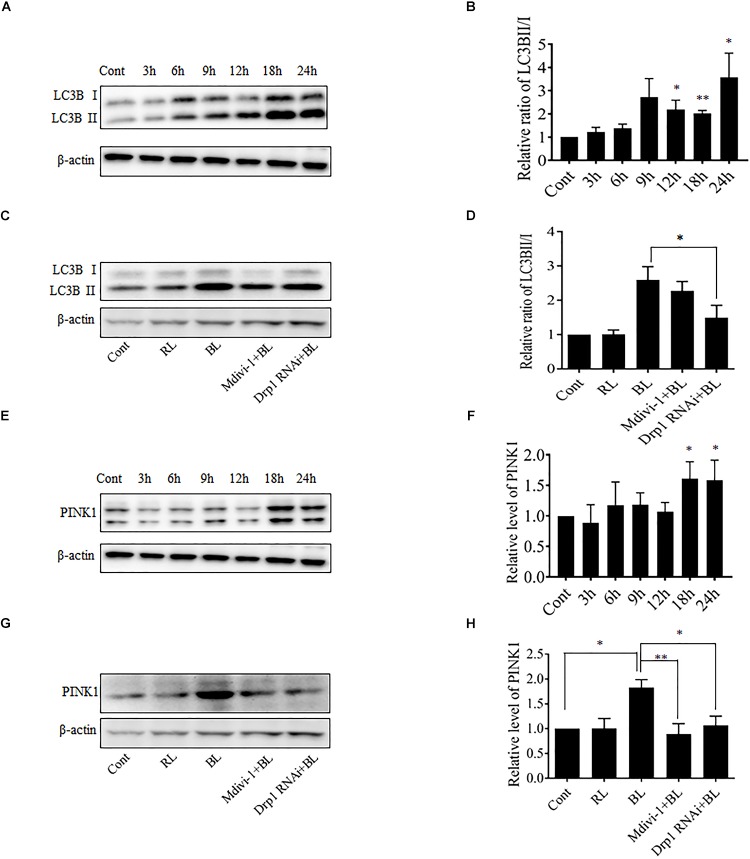
Effect of Mdivi-1 and Drp1 RNAi on LC3B lipidation and PINK1 expression after blue light irradiation. **(A,B)** After the indicated treatments, the levels of LC3BI and LC3BII were detected by western blot analysis. β-actin was used as an endogenous control. The graph indicates the normalized ratio of LC3BII/LC3BI in each group; ^∗^*p* < 0.05; ^∗∗^*p* < 0.01 vs. control. **(C,D)** Effect of Mdivi-1 and Drp1 RNAi on blue light-induced conversion of LC3BI to LC3BII. The graph indicates the normalized ratio of LC3BII/LC3BI in each group; ^∗^*p* < 0.05. **(E,F)** Expression level of PINK1 was detected by western blot analysis. The graph indicates the relative expression level of PINK1 in each group; ^∗^*p* < 0.05 vs. control. **(G,H)** Effect of Mdivi-1 and Drp1 RNAi on blue light-induced PINK1 expression. The graph indicates the relative expression level of PINK1 in each group; ^∗^*p* < 0.05; ^∗∗^*p* < 0.01. In all of the above graphs, the data are presented as the mean ± SD of four independent experiments. In contrast, no significant alteration was detected in LC3B lipidation and PINK1 expression in R28 cells after red light exposure (please refer to Supplementary Figures 1G–J).

**FIGURE 7:**
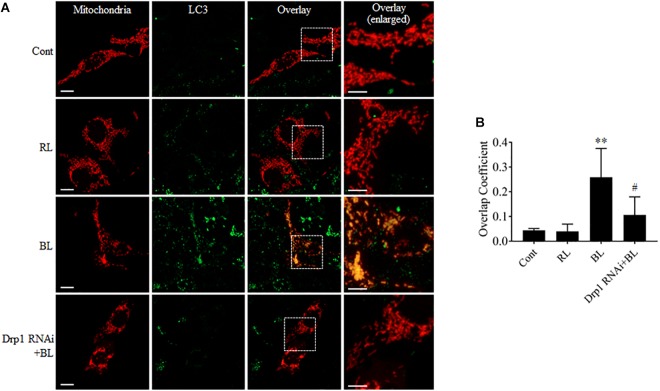
Mdivi-1 and Drp1 RNAi alleviated the co-localization of LC3 with mitochondria after blue light irradiation. **(A)** R28 Cells were transfected with pDsRed2-Mito to label mitochondria. The fluorescence signal of pDsRed2-Mito (red) indicates mitochondrial morphology. Green is the immunofluorescence signals of LC3, scale bar = 10 μm. The right panels show the co-localization of LC3 to mitochondria with higher magnification in the inserted boxes, scale bar = 5 μm. **(B)** The quantification of the co-localization of LC3 with mitochondria. Cellular outlines were traced on images and the overlap coefficient of co-localization between two fluorophores was measured using Image-Pro Plus 6.0 software. Co-localization values are represented as the mean and standard error from four independent experiments; ^#^*p* < 0.05 vs. BL. ^∗∗^*p* < 0.01 vs. control.

Excessive mitochondrial fission may disrupt mitochondrial functions, including oxidative phosphorylation, mitochondrial ROS production, and MMP (Park and Choi, 2012). In general, the damaged mitochondria are mostly removed by the process of mitophagy (Ni et al., 2015). Here, the conversion of autophagy marker LC3B and the expression level of mitophagy sensor PINK1 in R28 cells were examined using western blot analysis. It was found that the conversion of LC3B-I to LC3B-II was significantly enhanced after blue light irradiation for up to 12 h (Figures 6A,B). The expression of PINK1 was also significantly increased after blue light irradiation for up to 18 h (Figures 6E,F). In contrast, red light irradiation did not influence the conversion of LC3B-I to LC3B-II and PINK1 expression (Figures 6C,D,G,H and Supplementary Figures 1G–J). Drp1 RNAi efficiently alleviated blue light-induced PINK1 expression and LC3B conversion, whereas Mdivi-1 only attenuated blue light-induced PINK1 expression (Figures 6C,D,G,H). In addition, the immunofluorescence analysis of the co-localization of LC3 with mitochondria is a feasible approach to determine mitophagy using fluorescence microscopy. Here, it was found that much more fluorescent puncta (green) of LC3 were induced in R28 cells after blue light irradiation, and LC3 puncta were mostly co-localized with mitochondria. In contrast, few LC3 puncta co-localized with mitochondria were detected in cells exposed to red light or in the dark (control). Notably, Drp1 RNAi dramatically inhibited the co-localization of LC3 puncta with mitochondria (Figure 7). These results suggest the involvement of Drp1-mediated mitochondrial fission in blue light-induced mitophagy.

## Discussion

Light emitting diodes are widely used for general illumination. As a result, human eyes are exposed to ever-increasing levels of blue light emitted by LEDs. Several studies have indicated that blue light is phototoxic to the retina (Argun et al., 2014; Del Olmo-Aguado et al., 2016; Kim et al., 2016). Excessive exposure to blue light increases intracellular ROS and induces serious damage to photoreceptors and RGCs (Tosini et al., 2016). Furthermore, studies conducted by Osborne’s group indicate that blue light may damage RGCs by directly impinging on mitochondria (Yang et al., 2003; Osborne et al., 2014). It has been proposed that RGCs are more vulnerable to blue light than photoreceptors and retinal pigment epithelial cells (Del Olmo-Aguado et al., 2012). Recently, it has also been reported that light insult changes mitochondrial morphology in RGC-5 and R28 cells from elongated into the dot-like shape, which is similar to changes observed during mitochondrial fission (Knels et al., 2011; Del Olmo-Aguado et al., 2012). However, it is still unknown whether mitochondrial dynamics is involved in blue light-induced damage in RGCs. Here, we found that blue light significantly up-regulated the expression of mitochondrial fission protein Drp1. In contrast, mitochondrial fusion protein Mfn2 was down-regulated after blue light exposure for up to 12 h (Figure 1). Consistent with a previous study (Knels et al., 2011), mitochondria were changed from elongated, tubular, and thread-like networks to punctate dot-like shapes in R28 cells after blue light insult. Red light had little effect on mitochondrial morphology. Importantly, Drp1 inhibitor Mdivi-1 or knockdown of Drp1 by siRNA significantly alleviated blue light-induced mitochondrial fission and apoptosis in R28 cells (Figures 2A,B, 3). Moreover, the inhibitory effect of the antioxidant NAC on blue light-induced mitochondrial fission is much weaker than that of Mdivi-1 and Drp1 RNAi, although its antiapoptotic effect is similar to Mdivi-1 or Drp1 RNAi (Figures 2, 3). These data suggest the possible interaction between ROS and mitochondrial dynamics, as well as the critical role of ROS in blue light-induced apoptosis in R28 cells. Blue light irradiation might directly induce oxidative stress through its photochemical or photodynamic effect on mitochondrial chromophores (Tosini et al., 2016). Mitochondria are the target organelles of ROS as well as the major sources of ROS. As a result, neutralization of mitochondrial ROS by NAC modestly protected mitochondria against excessive fission after blue light irradiation (Figures 2A,B). In addition, excessive mitochondrial fission is also closely correlated to the increment of intercellular ROS (Yu et al., 2011). Therefore, inhibition of blue light-induced mitochondrial fission by Mdivi-1 or Drp1 RNAi significantly alleviated the increase in intracellular ROS and mitochondrial ROS (Figure 4). Furthermore, ROS are important regulators of the intrinsic apoptotic cascade in neurons (Franklin, 2011). Consistent with this, NAC had a potent anti-apoptotic effect on blue light-induced apoptosis in R28 cells similar to that of Mdivi-1 or Drp1 RNAi (Figure 3). The anti-apoptotic effect of NAC to some extent also implies the mitochondrial fission-independent mechanism in blue light-induced cellular damage. Taken together, these results show that blue light may stimulate mitochondrial fission through alterations in the expression of mitochondrial dynamics-related proteins and that mitochondrial fission is involved in blue light-induced apoptosis.

Moreover, mitochondrial dynamics are important for the maintenance of mitochondrial quantity and quality. Disruption of mitochondrial dynamics may damage mitochondrial functions, including ROS production, MMP, and oxidative phosphorylation. In this study, excessive mitochondrial fission was induced after blue light exposure for up to 12 h (Figures 2A,B). Consistent with previous studies (Wood et al., 2007; Sato et al., 2013), excessive fission after blue light exposure disrupted mitochondrial functions. The intracellular and mitochondrial ROS level increased significantly and MMP decreased dramatically. Interestingly, prevention of excessive mitochondrial fission by Mdivi-1 or Drp1 RNAi efficiently attenuated blue light-induced MMP decrease and ROS production (Figures 4, 5). It is well recognized that the breakdown of mitochondrial functions may facilitate activation of the mitochondria-associated intrinsic apoptosis pathway. In agreement with results reported by Wood and Funk (Osborne et al., 2008; Knels et al., 2011), we found that the cleaved caspase 3 was markedly increased after blue light irradiation. Inhibition of mitochondrial fission by Mdivi-1 or Drp1 RNAi efficiently alleviated blue light-induced cleavage of caspase 3 (Figures 3D,E). These results suggest that blue light insult disrupts mitochondrial functions via excessive mitochondrial fission and subsequently stimulates mitochondria-associated intrinsic apoptosis. However, other underlying mechanisms in blue light-induced cell damage cannot be excluded. Osborne and his colleagues found that blue light impinged on mitochondria in RGC-5 cells and induced cell death by caspase-independent necroptosis (Del Olmo-Aguado et al., 2016). Moreover, NF-κB p65 depletion and COX III up-regulation in mitochondria also trigger apoptosis in the retina after light irradiation (Tomita et al., 2016). In fact, crosstalk occurs among the pathways of apoptosis, necroptosis, and necrosis. Thus, there is a possibility that mitochondria act as a crucial mediator in the crosstalk between apoptosis and necroptosis induced by blue light.

Mitochondria are important organelles that provide ATP and many nutrients for eukaryotic cells. However, the damaged mitochondria exert a negative influence on cellular survival. To maintain mitochondrial homeostasis, there are some important quality control systems to remove the irreversibly damaged mitochondria in cells (Ni et al., 2015; Rüb et al., 2017; Shi et al., 2018). Among them, the PINK1/parkin-mediated mitophagy plays a prominent role in the removal of damaged mitochondria. Mitochondrial fission also serves to specifically remove individual damaged mitochondria (Youle and van der Bliek, 2012). It is well recognized that excessive fission may lead to mitochondrial defects, including disruption of MMP and increased ROS production. Subsequently, MMP disruption and increased ROS can activate PINK1/parkin-mediated mitophagy (Priyadarshini et al., 2013; Rüb et al., 2017). Furthermore, the PINK1/parkin-mediated ubiquitination facilitates the degradation of mitofusins, which contributes to mitochondrial fission (Gegg et al., 2010; Tanaka et al., 2010). Thus, there is a close connection between mitochondrial dynamics and mitophagy (Ni et al., 2015). In agreement with this, we found that blue light irradiation enhanced the expression of mitophagy sensor PINK1 and conversion of autophagy marker LC3B (Figure 6) and stimulated the co-localization of LC3 with mitochondria (Figure 7). LC3 lipidation and co-localization of LC3 with mitochondria are considered as a crucial step in the maturation of autophagosomes and the occurrence of mitophagy, respectively (Kabeya et al., 2000; Ding and Yin, 2012). Importantly, Mdivi-1 or Drp1 RNAi efficiently alleviated the alterations in the levels of Mfn2, PINK1 and the co-localization of LC3 with mitochondria after blue light exposure (Figures 2, 6, 7). Although Mdivi-1 cannot directly inhibit the process of mitophagy, it did alleviate the blue light-induced mitochondrial fission by inhibiting the GTPase activity of Drp1. Thus, Mdivi-1 and Drp1 RNAi protect mitochondria against irreversible damage and subsequently attenuate the PINK1/parkin-mediated mitophagy after irradiation. Moreover, prevention of blue light-induced PINK1 up-regulation by Mdivi-1 or Drp1 RNAi (Figures 6G,H) can also reduce the ubiquitination and degradation of Mfn2. These results suggest that blue light-induced mitochondrial dynamics and mitophagy are closely linked.

**FIGURE 8:**
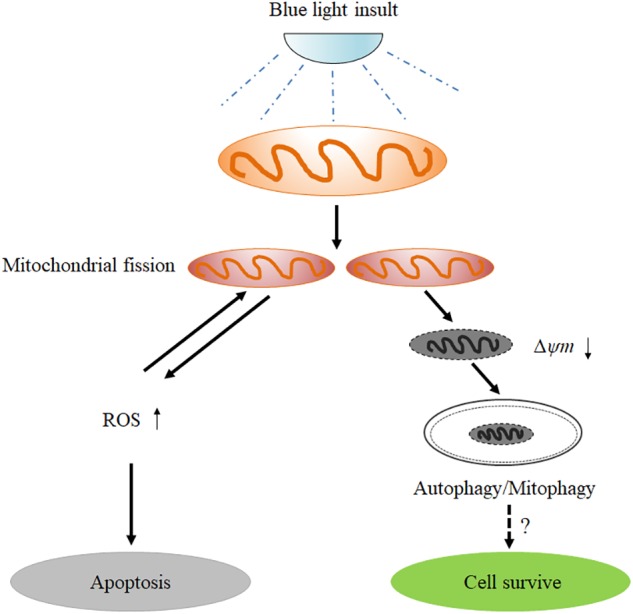
Schematic figure showing the role of mitochondrial fission in blue light-induced apoptosis in retinal neuronal R28 cells. Blue light insult stimulates mitochondrial fission through the alterations in mitochondrial dynamics-related proteins. The excessive fission disrupts mitochondrial functions, thereby enhancing the intracellular ROS production and decreasing mitochondrial membrane potential. The increased ROS induces cellular apoptosis or conversely impinges on mitochondrial dynamics. Furthermore, the mitochondria with disrupted mitochondrial membrane potential after blue light exposure are removed by mitophagy, although its role in cellular survival remains to be elucidated.

In conclusion, the results obtained in the present study provide important new insights into the understanding of the role of mitochondrial dynamics in blue light-induced apoptosis in RGCs (Figure 8). Blue light stimulates mitochondrial fission through alterations in mitochondrial dynamics-related proteins. The excessive fission disrupts mitochondrial functions, thereby enhancing the intracellular production of ROS and disrupting MMP. Increased ROS subsequently induces cellular apoptosis. Moreover, the disruption of MMP activates PIINK1/parkin-mediated mitophagy. Inhibition of mitochondrial fission by Mdivi-1 or Drp1 RNAi efficiently alleviates blue light-induced apoptosis and mitophagy in R28 cells, although the role of mitophagy in cellular survival remains to be elucidated.

## Author Contributions

X-JH conceived and designed the research. J-YL, KZ, DX, J-XT, and W-TZ conducted the cellular and molecular biological experiments. Y-YW, D-DY, M-YG, W-CZ, and L-PJ examined the mitochondrial morphology and functions. W-QF and FY constructed the blue and red light irradiation devices and monitored the spectral parameters. X-JH, J-YL, and KZ wrote the manuscript. J-XT and W-CZ participated in the majority of the supplemental experiments, experimental data analysis, manuscript revisions, and responses to reviewers’ recommendations. All authors analyzed the data and have given approval to the final version of the manuscript.

## Conflict of Interest Statement

The authors declare that the research was conducted in the absence of any commercial or financial relationships that could be construed as a potential conflict of interest.
