# Supplementary material for: Mitochondrial Fission Is Required for Blue Light-Induced Apoptosis and Mitophagy in Retinal Neuronal R28 Cells
Source: Front Mol Neurosci. 2018 Nov 27;11:432. doi: 10.3389/fnmol.2018.00432 (PMC6277708; doi:10.3389/fnmol.2018.00432)
Supplement: Supplementary file 1 [file Data_Sheet_1.pdf]

Supplementary Figures

Mitochondrial fission is required for blue light-induced apoptosis in retinal neuronal R28 cells

Jia-Yu Li [1†], Kun Zhang [1†], Dan Xu [1†], Wen-Tian Zhou [1†], Wen-Qing Fang [2], Yu-Ying Wan [3], Dan-Dan Yan [4], Miao-Yu Guo [1], Jin-Xin Tao [1], Wen-Chuan Zhou [1], Fan Yang [2], Li-Ping Jiang [4] and Xiao-Jian Han [1]*

*** Correspondence:** Xiao-Jian Han: hanxiaojian@hotmail.com

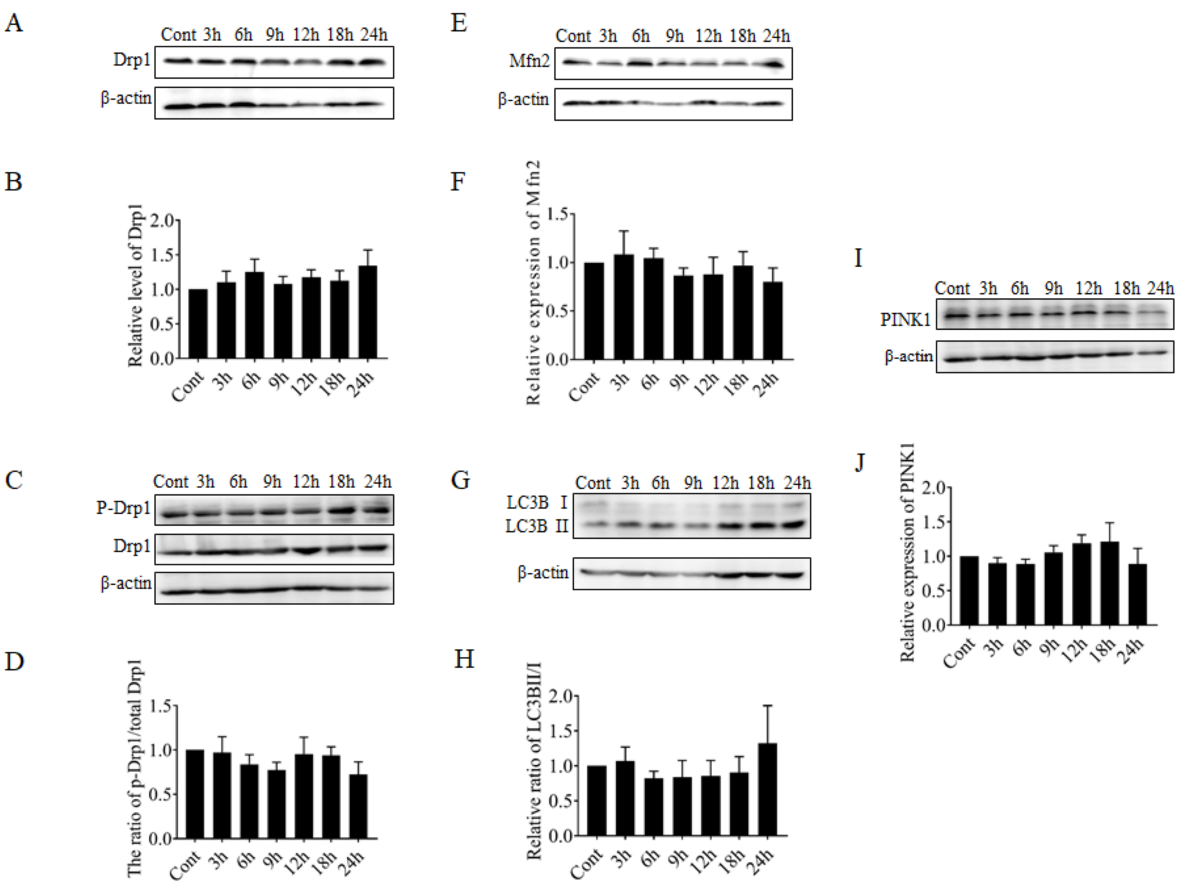

Suppl Fig. 1

Supplementary Figure 1. Effect of red light on expression of mitochondrial dynamics-related proteins and mitophagy-related proteins.

(**A**, **C**, **E**) After red light irradiation for the indicated durations, R28 cells were harvested for western blot analysis to detect the level of Drp1 (**A**), phosph-Drp1 at Ser616 (**C**), and Mfn2 (**E**). Cells cultured in the dark were used as controls. β-actin was used as an endogenous control. (**B**, **D**, **F**) The graphs indicate the relative levels of Drp1 (**B**), phosph-Drp1 (**D**), and Mfn2 (**F**) in cells after red light exposure at indicated time points. Relative level of each protein was indicated as a normalization of the ratio of mitochondrial dynamics-related protein/β-actin in each sample to control. All data are presented as the mean ± SD of four independent experiments. Please refer to Fig. 1 for the effect of blue light on mitochondrial dynamics-related proteins. (**G-J**) Effect of red light on expression of mitophagy-related proteins. After red light irradiation for the indicated durations, the level of LC3BII/I (**G**) and PINK1 (**I**) was detected by western blot. Cells cultured in the dark were used as controls. β-actin was used as an endogenous control. The graph indicates the normalized ratio of LC3BII/LC3BI (**H**) and relative level of PINK1 (**J**) in each group. Data are presented as the means ± SD of four independent experiments. Please refer to Fig. 6 for the effect of blue light on mitophagy-related proteins.

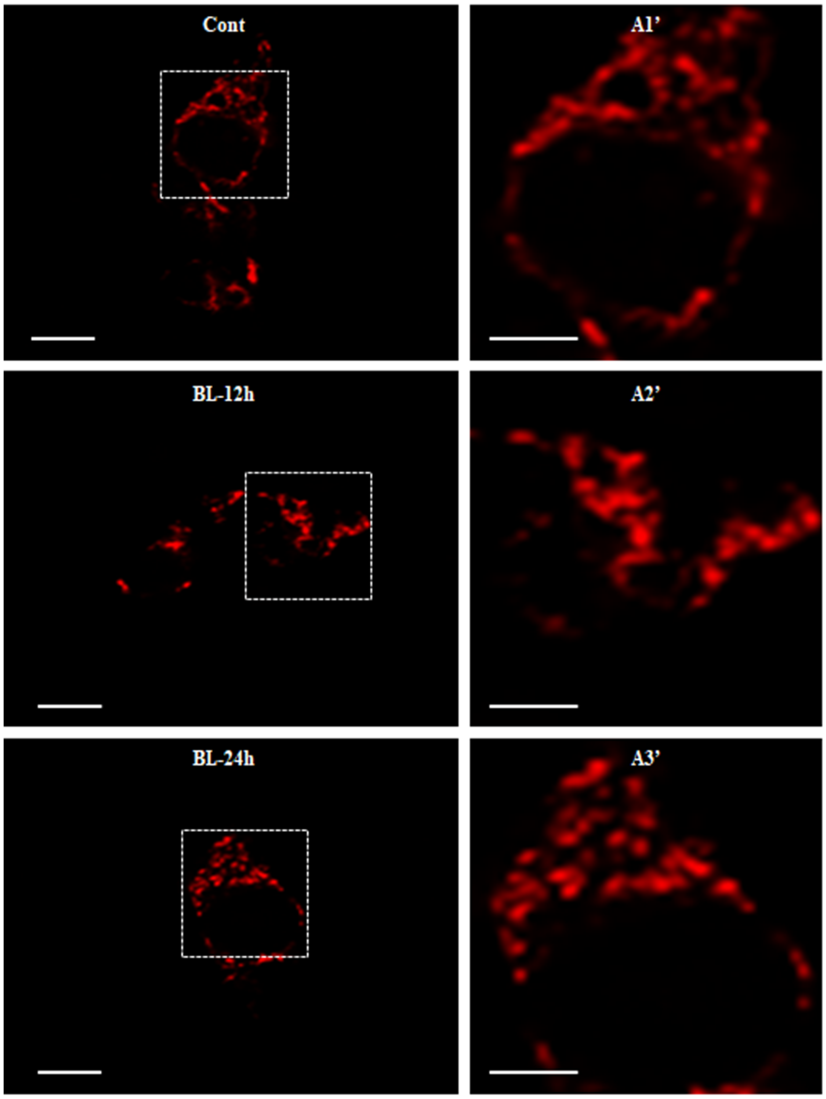

Suppl Fig. 2

Supplementary Figure 2. The fluorescence of mitochondria-targeted DsRed was highly tolerant to prolonged blue light irradiation.

R28 cells were transfected with pDsRed2-Mito to label mitochondria and cultured in the dark (control) or exposed to blue light for 12 or 24 h. The fluorescence signals in cells with or without irradiation were detected under microscope, scale bar = 10 µm. A1', A2' and A3' show the fluorescence signals with higher magnification in the inserted boxes, scale bar = 5 µm. Please refer to Fig. 2 for the effect of blue light on mitochondrial dynamics.

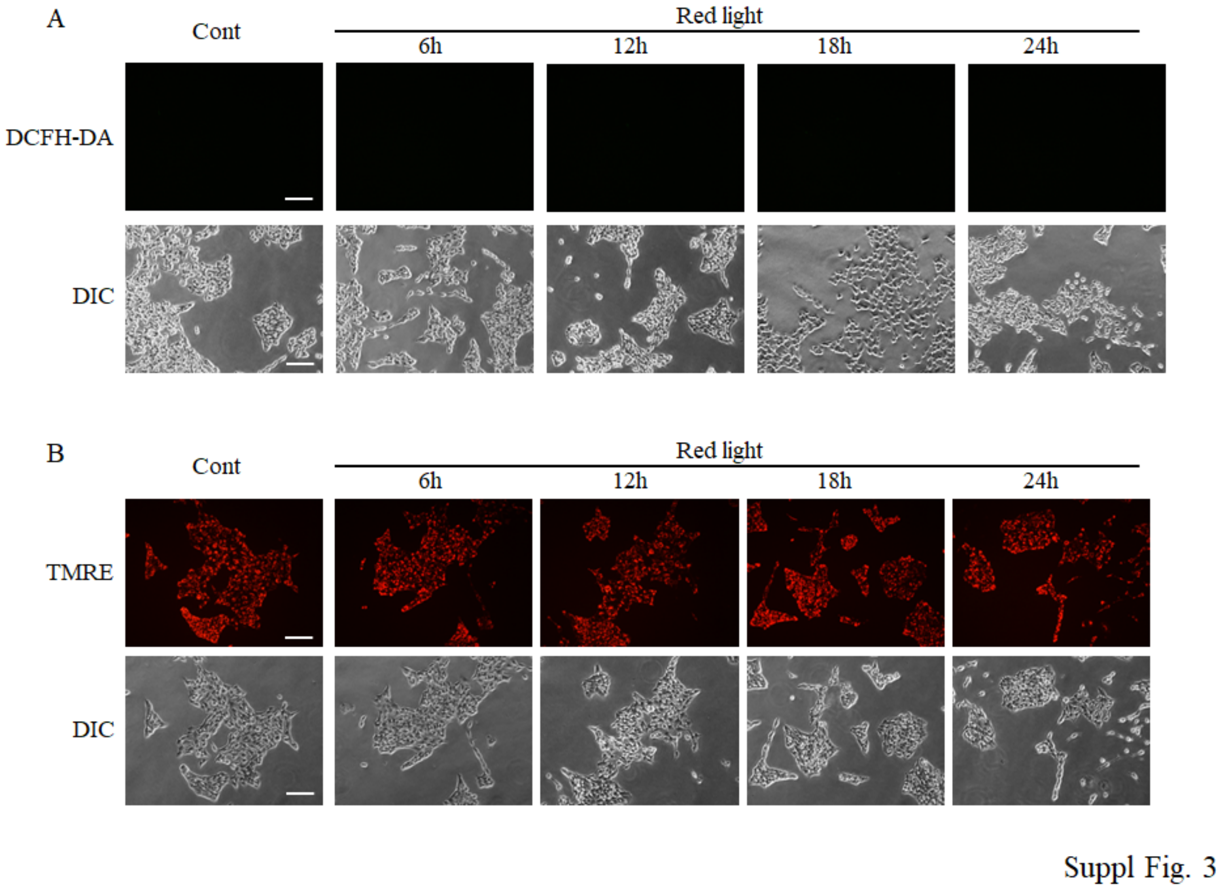

Supplementary Figure 3. Red light irradiation did not affect the intracellular ROS production and mitochondrial membrane potential.

(**A**) R28 cells were cultured in the dark (control) or exposed to red light for the indicated duration. The fluorescence signals of DCFH-DA were detected as the intracellular ROS under a fluorescence microscope. Scale bar = 50 μm. (**B**) R28 cells were cultured in the dark (control) or exposed to red light for the indicated duration. The fluorescence signals of TMRE were detected as mitochondrial membrane potential under a fluorescence microscope. Scale bar = 50 μm. Please refer to Fig. 4-5 for the effect of blue light on intracellular ROS and mitochondrial membrane potential.

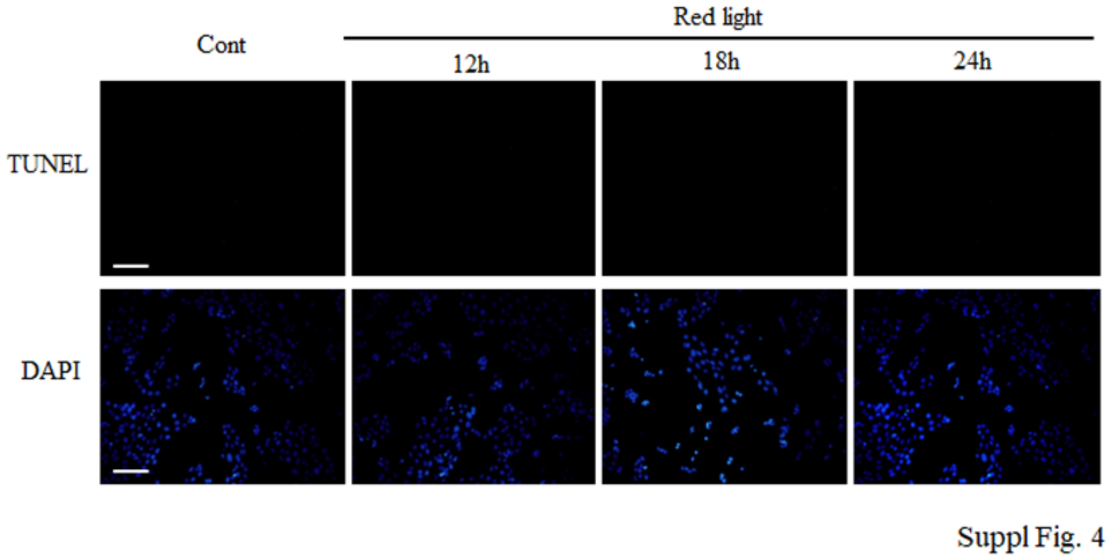

Supplementary Figure 4. Effect of red light on the survival of R28 cells.

After red light irradiation for the indicated periods, R28 cells were stained with TUNEL to detect cellular apoptosis and counterstained with DAPI. Cells cultured in the dark were used as controls. Scale bar = 50 μm. Please refer to Fig. 3 for the effect of blue light on the survival of R28 cells.
